# Implementation and Performance of First‐Trimester Referral Ultrasound Scan Following the Introduction of National Guidelines

**DOI:** 10.1002/jcu.70181

**Published:** 2026-02-05

**Authors:** Grazia Volpe, Laura Sarno, Elena Mantovani, Daniele Di Mascio, Valentina D'Ambrosio, Tiziana Fanelli, Ilaria Fantasia, Gian Piero Minnella, Paola Quaresima, Enrico Corno, Andrea Dall'Asta, the SIEOGiovani Committee of the Italian Society of Ultrasound in Obstetrics and Gynecology (SIEOG)

**Affiliations:** ^1^ Prenatal Diagnosis and Fetal Surgery Unit Fondazione IRCCS Ca' Granda, Ospedale Maggiore Policlinico Milan Italy; ^2^ Department of Neuroscience, Reproductive Sciences and Dentistry, School of Medicine University of Naples Federico II Naples Italy; ^3^ Department of Obstetrics and Gynecology Azienda Ospedaliera Universitaria Integrata Verona Verona Italy; ^4^ Department of Maternal and Child Health and Urological Sciences, Umberto I Hospital Sapienza University of Rome Rome Italy; ^5^ Fetal Medicine Unit Di Venere and Sarcone Hospitals Bari Italy; ^6^ Obstetrics and Gynecology Unit San Salvatore Hospital L'Aquila Italy; ^7^ Department of Obstetrics and Gynecology Azienda Ospedaliera Universitaria Policlinico Paolo Giaccone Palermo Italy; ^8^ Department of Obstetrics and Gynaecology Magna Graecia University of Catanzaro Catanzaro Italy; ^9^ Department of Medicine and Surgery, Obstetrics and Gynecology Unit University of Parma Parma Italy

**Keywords:** anatomy scan, congenital anomaly, early pregnancy, first trimester, prenatal diagnosis, ultrasound

## Abstract

**Background:**

To report the implementation across Fetal Medicine units and the agreement between first and second trimester referral scans in the identification of fetal anomalies in cases referred for the expert assessment of the fetal anatomy in the first trimester following the publication of the national guidelines in Italy.

**Methods:**

This multicenter, retrospective study aimed to evaluate the implementation and diagnostic performance of first‐trimester referral ultrasound (US) in identifying fetal anomalies, following the introduction of national guidelines in Italy. The analysis included singleton pregnancies referred to nine specialized Fetal Medicine centers between 11^+0^ and 13^+6^ weeks' gestation due to increased risk for structural anomalies. Risk was defined as either a nuchal translucency (NT) measurement ≥ 3.5 mm or suspicion of a structural anomaly at initial screening. Only cases undergoing referral US within the specified gestational window were included. Diagnostic accuracy was assessed by comparing findings from first‐trimester referral US with those from follow‐up referral US performed either at 14–16 weeks or at 19–21 weeks.

**Results:**

Out of 344 referred cases, 322 (93.6%) underwent first‐trimester referral US within the appropriate timeframe. After excluding miscarriages and terminations, 136 cases were evaluated again at 19–21 weeks and 207 at 14–16 weeks. The agreement between the 11^+0^–13^+6^ week and 19–21 week scans was 85.3%, with a sensitivity of 82.0%, specificity of 88.0%, positive predictive value (PPV) of 84.7%, and negative predictive value (NPV) of 85.7%. Agreement between the early and 14–16 week scans was 91.3%, with sensitivity, specificity, PPV, and NPV of 90.7%, 92.1%, 93.9%, and 88.2%, respectively.

**Conclusion:**

The study demonstrates successful implementation of national first‐trimester referral US guidelines in Italy. When performed by experienced operators using a standardized protocol, first‐trimester anatomy assessment is feasible and provides high diagnostic accuracy, enabling early detection of structural fetal anomalies.

## Graphical abstract

The expert assessment of fetal anatomy before 14 weeks is feasible when adopting a standardized protocol and allows an early diagnosis in most cases at risk for fetal anomaly following first trimester screening ultrasound.

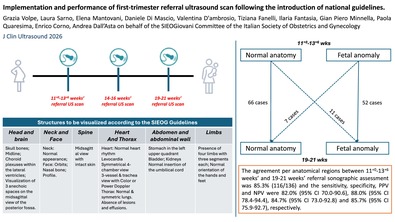

## 1 Introduction

First trimester ultrasound has been widely recommended by national and international guidelines to assess pregnancy viability and dating, determine the number of gestational sacs and fetuses, and screen for aneuploidies and preeclampsia (Nicolaides 2003; Nicolaides et al. 1992; Rolnik, Wright, Poon, O'Gorman, et al. 2017; Kagan et al. 2008; Bilardo et al. 2023; Italian Society For Ultrasound in Obstetrics and Gynecology (SIEOG) 2022; *Journal of Ultrasound in Medicine* 2021), the latter being performed between 11^+0^ and 13^+6^ weeks (Volpe et al. 2022). Even though the screening of fetal anomalies (Rolnik, Wright, Poon, Syngelaki, et al. 2017; Salomon et al. 2022) as well as the expert assessment of fetal anatomy are recommended in the second trimester (Carvalho et al. 2023; De Robertis et al. 2022), an early anatomy assessment during first trimester ultrasound at 11^+0^–13^+6^ weeks is also increasingly performed to evaluate the fetal anatomy, aiming to anticipate the detection of congenital anomalies and improve counseling and management.

Several studies have focused on the performance of first trimester anatomy screening in identifying fetal abnormalities, with different detection rates varying depending upon the setting (i.e., high‐ vs. low‐risk), the anatomical region evaluated or the type of anomaly (De Robertis and Italian Society of Ultrasound in Obstetrics and Gynecology (SIEOG) Working Group on the Obstetric Referral Scan 2023; Syngelaki et al. 2011). In this scenario, a meta‐analysis demonstrated an overall detection rate for fetal anomalies at 11^+0^–13^+6^ weeks of 32% in an unselected population and 61% in the context of a high‐risk setting. Of note, such detection rate has been shown to increase when standardized protocols for the assessment of the fetal anatomy are implemented (Karim et al. 2017). This is consistent with the latest version of the guidelines of the International Society of Ultrasound in Obstetrics and Gynecology (ISUOG) on the performance of 11^+0^–13^+6^ weeks scan, which include a list of anatomical regions and structures to be visualized for the assessment of fetal anatomy in a screening and in an expert setting as well as the structures that can be visualized in a detailed anatomical survey performed in the first trimester (Bilardo et al. 2023).

In 2021, the Italian Society of Ultrasound in Obstetrics and Gynecology (SIEOG) issued the updated version of the national guidelines regulating the obstetrical and gynecological ultrasound practice in the Italian territory (Italian Society For Ultrasound in Obstetrics and Gynecology (SIEOG) 2022). With respect to first trimester ultrasound, these guidelines recommend the referral to a Fetal Medicine unit for the expert assessment of fetal anatomy and provide a dedicated, standardized protocol, for early anatomy scan of all cases identified at risk for structural anomaly following first trimester screening ultrasound. The aim of this study was to report the implementation across Fetal Medicine units and the agreement between first and second trimester referral scans in the identification of fetal anomalies in cases referred for the expert assessment of the fetal anatomy in the first trimester following the publication of the SIEOG Guidelines.

## 2 Methods

### 2.1 Study Design and Participants

This was a multicenter, retrospective study of prospectively collected data conducted in nine Fetal Medicine units across Northern (University of Parma, University of Milan, University Hospital of Verona), Central (Sapienza University of Rome and San Salvatore Hospital, L'Aquila) and Southern (Federico II University of Naples, Magna Graecia University of Catanzaro, Di Venere and Sarcone Hospitals, Bari and Paolo Giaccone University Hospital, Palermo) Italy which included a consecutive series of singleton pregnancies identified at risk for structural anomaly following first trimester screening ultrasound referred between 1 December 2021 and 31 January 2023. As per SIEOG Guidelines, the definition of “high‐risk for anomaly” was based on either nuchal translucency ≥ 3.5 mm or one or more fetal structural anomalies suspected at first trimester screening ultrasound (Italian Society For Ultrasound in Obstetrics and Gynecology (SIEOG) 2022).

Only cases undergoing referral ultrasound at one of the participating Fetal Medicine Units between 11^+0^ and 13^+6^ weeks of gestation were included in the study. In such cases, the expert ultrasound assessment was performed according to the standardized protocol for fetuses at high risk for structural anomaly recommended by SIEOG (Società Italiana di Ecografia Ostetrica e Ginecologia e Metodologie Biofisiche 2024), which consists in a list of anatomical regions and structures to be visualized in the context of the expert assessment of fetal anatomy at 11^+0^–13^+6^ weeks (Table 1). In cases identified at high risk for anomaly in the first trimester, the SIEOG Guidelines also recommend referral second trimester fetal anomaly ultrasound assessment to be performed at Fetal Medicine unit if the pregnancy continues. Such a recommendation has been incorporated in the local protocol of the participating units in terms of early referral anomaly scan to be systematically performed at 14–16 weeks' gestation and followed by referral anomaly scan at 19–21 weeks' gestation. For the purposes of the study, only cases undergoing at least one referral ultrasound assessment beyond 13^+6^ weeks were eligible for enrolment.

**TABLE 1:** Anatomical structures to be visualized on detailed first trimester fetal scan according to the high‐risk protocol for first trimester assessment recommended by the Italian Society of Ultrasound in Obstetrics and Gynecology ((SIEOG) 2024; Karim et al. 2017), and comparison with protocols proposed by the American Institute of Ultrasound in Medicine (AIUM) (*Journal of Ultrasound in Medicine* 2021), the International Society of Ultrasound in Obstetrics and Gynecology (ISUOG) (Bilardo et al. 2023) and the World Association of Perinatal Medicine (WAPM) (Volpe et al. 2022) protocols.

Anatomical region	Structures to be visualized			
	SIEOG	AIUM	ISUOG	WAPM
Head and brain	Skull bones; Midline; Choroid plexuses within the lateral ventricles Visualization of 3 anechoic spaces on the midsagittal view of the posterior fossa	*Axial plane*: Skull bones, Midline, Choroid plexuses, Thalami, Posterior fossa, Ventricles^a^, Cortex^a^, Third Ventricle^a^ *Sagittal plane*: thalami, mid‐brain, brain stem, fourth ventricle, cisterna magna	Calcification and shape of the skull; Midline; Choroid plexuses; Thalami; Brainstem; Cerebral peduncles with aqueduct of Sylvius; Fourth ventricle Cisterna magna.	Skull; Midline falx; Lateral ventricles/CP; Symmetric, filled by CP Ventriculomegaly Cranial posterior fossa
Neck	Normal appearance	Normal appearance	Normal appearance	Normal appearance
Face	Orbits; Nasal bone; Profile	Orbits^a^; Nasal Bone; Profile; Maxilla; Mandible; Retronasal triangle with ancillary bones^a^	Forehead; Orbits; Nasal Bone; Maxilla; Retronasal triangle; Upper lip Mandible	Profile; Orbits, anterior palate
Spine	Midsagittal view with intact skin	Vertebral elements/alignment; Skin edge scapula^a^	Regular shape and continuity of spine	Vertebrae; Dorsal skin
Thorax	Normal and symmetric lungs Absence of lesions and effusions	Symmetric lungs; Ribs with normal shape, length, and ossification^a^ Diaphragm demarcation	Shape of the thoracic wall; Lung fields Diaphragmatic continuity	Lung fields
Heart	Normal heart rhythm Levocardia Symmetrical 4‐chamber view 3‐vessel and trachea view with Color or Power Doppler	Cardiac position and axis Angle measurement^a^ 4‐chamber view without Color 3‐vessel and trachea view with color Tricuspid valve flow^a^ Sagittal view of aortic and ductal arch^a^	Normal heart rhythm Situs Position, axis, and size 4‐chamber view with two distinct ventricles on grayscale and color Doppler Left ventricular outflow tract view on grayscale or color Doppler Three‐vessel‐and‐trachea view on grayscale or color Doppler Tricuspid and Ductus venosus flow on pulsed wave Doppler	Heart activity; Cardiac situs; Size and position; Four chambers; Three vessels/arches
Abdomen	Stomach in the left upper quadrant Bladder; Kidneys	Stomach; Liver; Portal vein^a^; Bladder Color Doppler of umbilical arteries on each side of the bladder; Ductus venosus flow^a^; kidneys	Stomach; Bladder; Kidneys; Two umbilical arteries bordering bladder	Stomach; Bladder; Kidneys; Two umbilical arteries bordering bladder Genital tubercle
Abdominal wall	Normal insertion of the umbilical cord	Normal insertion of the umbilical cord Contour of anterior wall	Intact abdominal wall with umbilical cord insertion	Umbilical cord insertion
Extremities	Presence of four limbs with three segments each; Normal orientation of the hands and feet	Presence of the 4 extremities; Presence of 3 long bones in each extremity and normal appearance Presence of hands/feet; Fingers/thumb/toes^a^; 3‐dimensional assessment of extremities^a^	Upper limbs with three segment and free movement; Lower limbs with three segments and free movement	Active movements, Three segments and Hands/feet

^a^ Only if required or suspicious.

Anomalies were counted by assuming that a positive result consisted in either the diagnosis or the suspicion of an anomaly or the finding of an anatomical anomaly of undetermined significance affecting at least one anatomical region or structure. This allowed to account for the fact that a diagnosis, a suspicion, or a finding of undetermined significance may be modified later in pregnancy as the anomaly initially identified or suspected in the first trimester may evolve or be reclassified (De Robertis and Italian Society of Ultrasound in Obstetrics and Gynecology (SIEOG) Working Group on the Obstetric Referral Scan 2023). Therefore, a “true” positive result was assigned if the anomaly diagnosed in the second trimester affected the same anatomical region or structure as that diagnosed or suspected at 11^+0^–13^+6^ weeks. Manual counting of anomalies affecting each anatomical region was undertaken and recorded separately between the expert ultrasound assessments performed at 11^+0^–13^+6^ weeks and at later gestation timeframes, that is, at 14–16 and 19–21 weeks. Consistently, false positive cases were defined if the diagnosis or suspicion of an anomaly or the finding of an anatomical anomaly of undetermined significance affecting one anatomical region at 11^+0^–13^+6^ weeks was not confirmed at expert ultrasound assessment performed later in gestation. False negatives were defined in the event of fetal anomaly not suspected or diagnosed in the first trimester but diagnosed at second trimester referral scan. “True” false negatives featuring anomalies always or potentially detectable in the first trimester were distinguished from “timely” false negatives—that is, cases showing evolving anomalies never detectable in the first trimester. Consistently, cases of fetal anomaly suspected or diagnosed in the first trimester but not confirmed at second trimester referral scan were classified as false positives. Among them, “true” false positives were defined in the event of a non‐evolving anomaly suspected or diagnosed in the first trimester that was not confirmed at later gestation, and were distinguished from “timely” false positives, which consisted in cases featuring evolving anomalies that can be diagnosed in the first trimester and resolve at later gestation (Syngelaki et al. 2011).

All ultrasound examinations were performed by Fetal Medicine specialists with dedicated expertise in prenatal diagnosis and early ultrasound assessment of the fetal anatomy using high‐end equipment, and in all the included cases fetal echocardiography was also carried out in accordance with SIEOG Guidelines (Società Italiana di Ecografia Ostetrica e Ginecologia e Metodologie Biofisiche 2024; Italian Society For Ultrasound in Obstetrics and Gynecology (SIEOG) 2022; De Robertis et al. 2022). Across the participating units, the time allocated for the expert ultrasound examinations in the first and second trimester was 40 min.

Miscarriage and termination of pregnancy within 13^+6^ weeks of gestation, as well as the absence of at least one ultrasound assessment performed in the second trimester, represented exclusion criteria for the study.

### 2.2 Outcomes

The primary aims of the study were to evaluate the implementation of first trimester referral ultrasound assessment across the participating Fetal Medicine units and its accuracy in discriminating between anomalous and non‐anomalous fetuses and, if anomalous, in identifying the affected anatomical regions or structures compared with mid‐trimester (i.e., 19–21 weeks') referral ultrasound. The secondary outcome was to compare such accuracy between first trimester and early second trimester (i.e., 14–16 weeks') referral ultrasound.

### 2.3 Statistical Analysis

Data on pregnancy outcome were collected from computerized records, while the prenatal information was recorded in fetal databases. Data were recorded and stored in a Microsoft Excel (Microsoft, Redmond, WA) secured pseudonymized database, which was accessible only by the members of the research team.

Statistical analysis was performed using IBM SPSS Statistic Version 28. Data were shown as mean ± standard deviation, median (range) or as number (percentage) as appropriate. The study was approved by the local ethics committee of the participating units and performed following the Strengthening the Reporting of Observational Studies in Epidemiology (STROBE) guidelines (Von Elm et al. 2014).

## 3 Results

### 3.1 Characteristics of the Included Cases

**FIGURE 1:**
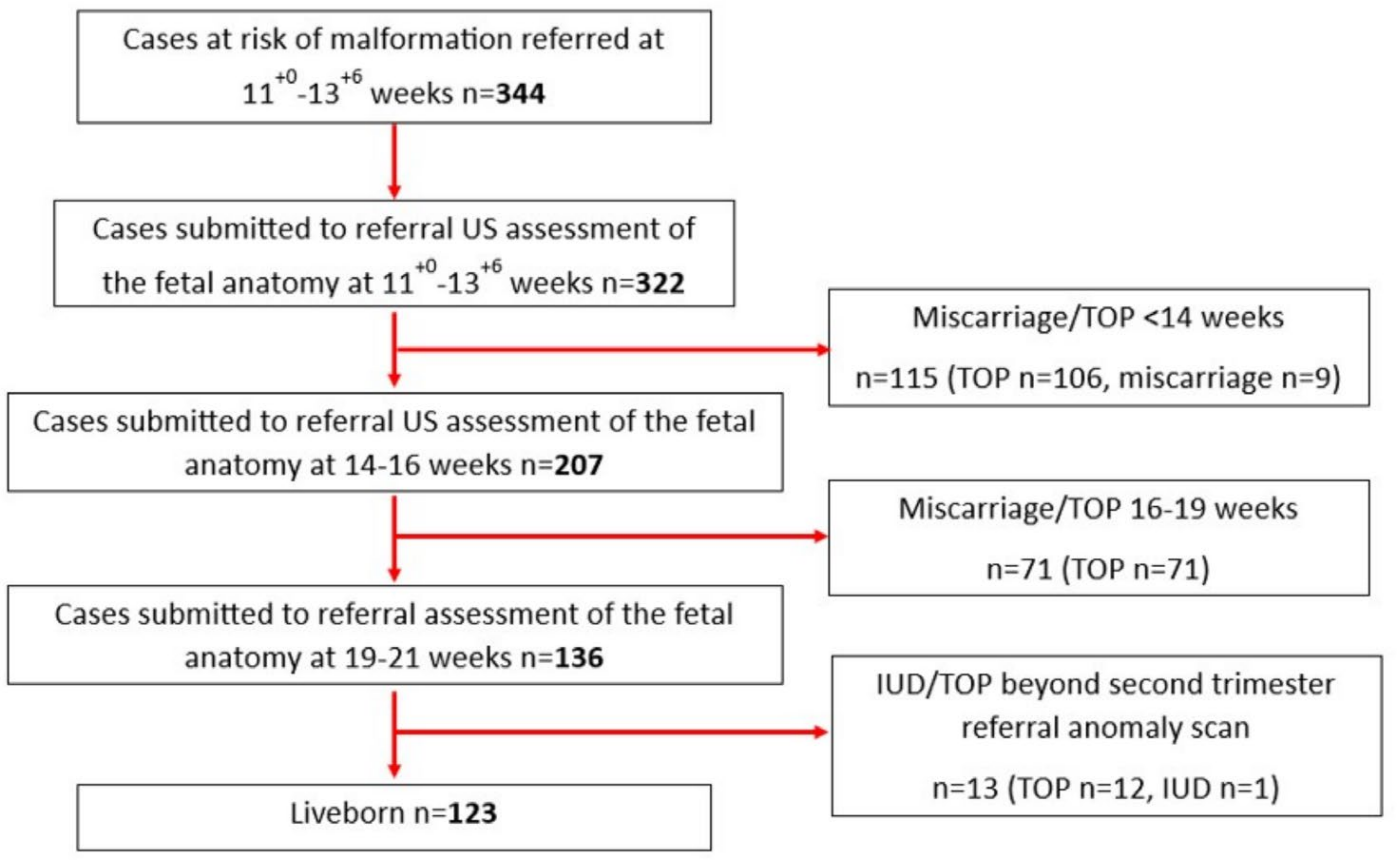
Flow chart (according to STROBE guidelines) for inclusion of cases. IUD, intrauterine death; TOP, termination of pregnancy; US, ultrasound.

Overall, 344 cases were referred between 11^+0^ and 13^+6^ weeks at the participating centers over the study period. Of these, 322 (93.6%) had referral sonographic assessment within 13^+6^ weeks of gestation, thus representing the study group. All the included patients were offered the option of invasive testing, which was accepted in 280 cases. Miscarriage/intrauterine death and termination of pregnancy (TOP) were recorded in 10 and 189 cases, respectively, as fetal loss was recorded in 115 cases prior to 14^+0^ weeks, in 71 prior to 19^+0^ weeks, and in 13 after mid‐trimester referral anomaly scan. Overall, 136 and 207 cases were available for the evaluation of the primary and the secondary outcomes, respectively (Figure 1). The baseline characteristics of the included cases are shown in Table 2.

**TABLE 2:** Features of the 136 cases having referral ultrasound scan at 19–21 weeks and of the 207 cases having referral ultrasound scan at 14–16 weeks.

Variable	Cases having referral ultrasound scan at 19–21 weeks; *N* 136	Cases having referral ultrasound scan at 14–16 weeks; *N* 207
Ethnicity, *N* (%)	Caucasian 128 (94.1%) African 5 (3.7%) Other 3 (2.2%)	Caucasian 191 (92.2%) African 8 (3.9%) Other 8 (3.9%)
Maternal age, years Mean ± SD	32.3 ± 6.2	33.4 ± 6.1
Nulliparity, *N* (%)	71 (52.2%)	104 (50.2%)
Booking BMI, kg/m^2^ Mean ± SD	25.7 ± 6.1	24.6 ± 4.5
Gestational age, weeks^+days^ Mean ± SD	12^+4^ ± 0^+5^	12^+4^ ± 0^+5^
Crown‐Rump Length at screening ultrasound scan, mm Mean ± SD	64.4 ± 8.9	63.2 ± 9.4
Nuchal translucency at screening ultrasound scan, mm Mean ± SD	2.8 ± 1.5	3.6 ± 2.2
Indication for referral first trimester scan, *N* (%)	NT ≥ 3.5 mm 58 (42.6%)	NT ≥ 3.5 mm 75 (36.2%)
	Suspected fetal anomaly 78 (57.4%)	Suspected fetal anomaly 132 (63.8%)
Invasive prenatal test, *N* (%)	107 (78.7%)	170 (82.1%)
Pathological results of invasive prenatal test, *N* (%)	12 (8.8%)	49 (23.7%)
Gestational age of first trimester referral ultrasound scan, weeks^+days^ Mean ± SD	13^+0^ ± 0^+4^	12^+6^ ± 0^+3^

### 3.2 Comparison of the Sonographic Findings in Pregnancies Continuing Until Mid‐Trimester Referral Anomaly Scan

The comparison of the sonographic findings across the different anatomical regions in the 136 cases having referral ultrasound at 11^+0^–13^+6^ weeks and 19–21 weeks is shown in Tables 3a and 3b. Of these, 77 had a non‐anomalous 11^+0^–13^+6^ weeks referral ultrasound. Normal sonographic findings at mid‐trimester were reported in 66 cases classified as non‐anomalous following referral sonographic assessment within 13^+6^ weeks' gestation. Among the cases classified as non‐anomalous there were 11 false negatives (Table 4). These included four cases of minor cardiac anomaly (i.e., three cases of ventricular septal defects and one case of persistent left superior vena cava) and one major cardiac anomaly (i.e., one case of atrioventricular septal defect); the other anomalies overlooked at 11^+0^–13^+6^ weeks included one case of ductus venous agenesis and one case of fibular hemimelia and clubfoot in association with ventricular septal defect. In the remaining four false negative diagnoses of first trimester referral ultrasound two cases of hydronephrosis, one case of agenesis of the corpus callosum and one case of fetal hydrops were recorded. There were also 5/11 (45.5%) cases diagnosed with multiple anomalies at 19–21 weeks following a diagnosis of single anomaly at 11^+0^–13^+6^ weeks. Four cases were classified as “timely false negatives,” namely one case of agenesis of the corpus callosum, one case of fetal hydrops and two cases of hydronephrosis.

**TABLE 3a:** Normal vs. abnormal sonographic findings across different anatomical regions in the 136 cases having referral ultrasound scan at 11^+0^–13^+6^ weeks and at 19–21 weeks.

Anatomical region	11^+0^–13^+6^ weeks' scan	19–21 weeks' scan
Head and brain	7	5
Neck	1	—
Face	2	1
Spine	1	—
Thorax	—	—
Heart	21	24
Abdomen	17	15
Abdominal wall	3	3
Extremities	4	3
Multiple anomalies	3	11
Normal	77	74

**TABLE 3b:** Normal vs. abnormal sonographic findings across different anatomical regions in the 207 cases having referral ultrasound scan at 11^+0^–13^+6^ weeks and at 14–16 weeks.

Anatomical region	11^+0^–13^+6^ weeks' scan	14–16 weeks' scan
Head and brain	15	13
Neck	1	1
Face	3	2
Spine	2	4
Thorax	2	2
Heart	36	39
Abdomen	23	19
Abdominal wall	8	5
Extremities	7	8
Multiple anomalies	16	25
Normal	94	89

**TABLE 4:** False negative diagnoses of 11^+0^–13^+6^ weeks' referral ultrasound with respect to early (14–16 weeks) and mid‐trimester (19–21 weeks) referral anomaly scan.

	Maternal age	parity	BMI	Ethnicity	CRL (mm)^a^	NT (mm)^a^	Prenatal invasive test	Referral scan at 11^+0^–13^+6^ weeks	Referral scan at 14–16 weeks	Referral scan at 19–21 weeks	Pregnancy outcome
*1*	29	0	23	Caucasian	61.5	4.9	Yes, normal	No anomalies	No anomalies	*Ventricular septal defect, hydronephrosis*	Delivery at term
*2*	37	1	26	African	80	4.3	Yes, trisomy 21	No anomalies	No anomalies	*Persistent left superior vena cava*	Delivery at term
*3*	38	0	22	Caucasian	72.6	6.2	Yes, normal	No anomalies	Ductus venous agenesis	*Ductus venous agenesis*	Delivery at term
*4*	44	0	21	Caucasian	72	3.6	Yes, normal	No anomalies	No anomalies	*Agenesis of the corpus callosum*	Delivery at term
*5*	41	1	22	Caucasian	55.5	4.1	Declined	No anomalies	Atrioventricular septal defect	*Atrioventricular septal defect*	Delivery at term
*6*	34	1	26	Caucasian	56.3	3.8	Yes, normal	No anomalies	Fibular hemimelia, talipes	*Fibular hemimelia, talipes, ventricular septal defect*	Termination of pregnancy
*7*	29	0	21	Caucasian	77.9	5.2	Yes, 22q11.21 deletion	No anomalies	No anomalies	*Fetal hydrops*	Delivery at term
*8*	39	3	22	Caucasian	55.2	4.2	Yes, trisomy 21	No anomalies	Hydronephrosis	*Hydronephrosis*	Delivery at term
*9*	30	1	18	Caucasian	76.5	3.5	Yes, trisomy 21	No anomalies	No anomalies	*Hydronephrosis*	Delivery at term
*10*	28	3	23	Caucasian	64	3.6	Yes, normal	No anomalies	No anomalies	*Ventricular septal defect*	Delivery at term
*11*	33	0	24	Asian	49	4	Yes, normal	No anomalies	No anomalies	*Ventricular septal defect*	Delivery at term
*12*	42	0	22	Caucasian	53.4	5.5	Yes, trisomy 22	No anomalies	Fetal tail	*—*	Termination of pregnancy
*13*	37	2	24	Caucasian	57.8	7.8	Yes, trisomy 13	No anomalies	Polydactyly, interrupted aortic arch	*—*	Termination of pregnancy
*14*	37	0	28	Caucasian	63	8.4	Yes, normal	No anomalies	Renal dysplasia, micrognathia	*—*	Termination of pregnancy
*15*	34	0	21	Caucasian	50	6	Yes, XX, del (10)(p11.2)	No anomalies	Double outlet right ventricle, fetal hydrops	*—*	Termination of pregnancy
*16*	40	0	27	Caucasian	64	4.7	Yes, trisomy 21	No anomalies	Ventricular septal defect	*—*	Termination of pregnancy
*17*	27	0	23	Caucasian	67.2	13.6	Yes, 45,X0	No anomalies	Hypoplastic left heart syndrome	*—*	Termination of pregnancy
*18*	32	2	29	Asian	57	4.9	Yes, trisomy 18	No anomalies	Ventricular septal defect	*—*	Termination of pregnancy

^a^ At screening ultrasound scan.

False positives of 11^+0^–13^+6^ weeks' scan were recorded in nine cases (Table 5). These included one case of tetralogy of Fallot, three cases of Blake's pouch cyst (two of which were diagnosed with a cardiac anomaly at 19–21 weeks) and one case of cleft lip. In the remaining four false positive diagnoses of first trimester referral ultrasound, one case of first trimester megacystis, one case of cervical lymphangioma, and two cases of abdominal cyst were recorded.

**TABLE 5:** False positive diagnoses of 11^+0^–13^+6^ weeks' referral ultrasound with respect to early (14–16 weeks) and mid‐trimester (19–21 weeks) referral anomaly scan.

	Maternal age	Parity	BMI	Ethnicity	CRL (mm)^a^	NT (mm)^a^	Prenatal invasive test	Referral scan at 11^+0^–13^+6^ weeks	Referral scan at 14–16 weeks	Referral scan at 19–21 weeks	Pregnancy outcome
*1*	36	0	23	Caucasian	54.4	1.9	Yes, normal	Megacystis	No anomalies	No anomalies	Delivery at term
*2*	21	0	21	Caucasian	64.1	3.4	Yes, normal	Cervical lymphangioma	Cervical lymphangioma	No anomalies	Delivery at term
*3*	40	1	35	Caucasian	50	2.0	Yes, normal	Cleft lip	No anomalies	No anomalies	Delivery at term
*4*	37	2	22	Caucasian	56	1.7	Declined	Abdominal cyst	No anomalies	No anomalies	Delivery at term
*5*	31	2	25	Caucasian	61.5	2.1	Declined	Abdominal cyst	No anomalies	No anomalies	Delivery at term
*6*	37	1	22	Caucasian	73	3.8	Yes, normal	Tetralogy of Fallot	No anomalies	No anomalies	Delivery at term
*7*	40	0	29	Caucasian	69	2.4	Yes, normal	Blake's pouch cyst	No anomalies	No anomalies	Delivery at term
*8*	38	1	23	Caucasian	83	2.3	Declined	Blake's pouch cyst	No anomalies	Ventricular septal defect	Delivery at term
*9*	32	0	29	Caucasian	58	1.4	Yes, 47,XY,+12	Blake's pouch cyst	Blake's pouch cyst	Coarctation of the aorta	Termination of pregnancy

^a^ At screening ultrasound scan.

Overall, the agreement per anatomical regions between 11^+0^–13^+6^ weeks' and 19–21 weeks' referral sonographic assessment was 85.3% (116/136) and the sensitivity, specificity, PPV, and NPV were 82.0% (95% CI 70.0–90.6), 88.0% (95% CI 78.4–94.4), 84.7% (95% CI 73.0–92.8), and 85.7% (95% CI 75.9–92.7), respectively.

Six cases were classified as “timely false positives”, namely one case of megacystis, one case of cervical lymphangioma, two cases of abdominal cyst and two cases of Blake's pouch cyst. After excluding timely false negatives and timely false positives, the agreement per anatomical regions between 11^+0^–13^+6^ weeks' and 19–21 weeks' referral sonographic assessment was 92.1% (116/126) and the sensitivity, specificity, PPV and NPV were 87.8% (95% CI 76.3–94.9), 95.7% (95% CI 87.8–99.1), 94.3% (95% CI 84.3–98.8), and 90.4% (95% CI 81.2–96.1), respectively.

### 3.3 Comparison of the Sonographic Findings in Pregnancies Continuing Until Early Referral Anomaly Scan

The comparison of the sonographic findings across the different anatomical regions in the 207 cases having referral ultrasound at 11^+0^–13^+6^ weeks and at 14–16 weeks is shown in Tables 3a and 3b. Of these, 93 had a non‐anomalous 11^+0^–13^+6^ weeks referral ultrasound. Normal sonographic findings at 14–16 weeks were reported in 82 cases classified as non‐anomalous following referral sonographic assessment within 13^+6^ weeks' gestation. False negatives of 11^+0^–13^+6^ compared with 14–16 weeks' referral scan were recorded in 11 cases (Table 4). Among these, minor cardiac anomalies were recorded in two cases (ventricular septal defects) and major cardiac anomalies in four (i.e., one case of atrioventricular septal defect, one case of interrupted aortic arch, one case of hypoplastic left heart syndrome and one case of double outlet right ventricle); the other anomalies overlooked at 11^+0^–13^+6^ weeks included one case of ductus venous agenesis, two cases of multiple anomalies (one case of fibular hemimelia and talipes, later found to be associated with ventricular septal defect; one case of micrognathia associated with renal dysplasia) and one case of fetal tail. In the remaining false negative case of first trimester referral ultrasound, hydronephrosis was recorded.

False positives of 11^+0^–13^+6^ weeks' referral scan were recorded in seven cases (Table 5). These included one case of tetralogy of Fallot, two cases of Blake's pouch cyst, and one case of cleft lip. In the remaining three false positive diagnoses of first trimester referral ultrasound, one case of megacystis and two cases of abdominal cyst were recorded. There was no case of non‐confirmed anomaly detected at 11^+0^–13^+6^ weeks showing a structural anomaly affecting a different anatomical structure or region at expert early anomaly scan.

Overall, an agreement between referral US scan at 11^+0^–13^+6^ weeks and referral anomaly scan at 14–16 weeks was recorded in 189/207 (91.3%) cases and sensitivity, specificity, PPV and NPV were 90.7% (95% CI 83.9–95.3), 92.1% (95% CI 84.5–96.8), 93.9% (95% CI 87.8–97.5), and 88.2% (95% CI 79.8–94.0), respectively.

## 4 Discussion

### 4.1 Key Findings

This is the first study reporting data on a case series of first‐trimester expert ultrasound across referral Fetal Medicine units following the publication of guidelines regulating the obstetric ultrasound practice on a national scale. Our findings show excellent implementation of the guidelines for first trimester referral ultrasound across the referral units participating in the study in the 14 months following their publication. Furthermore, in our population at high‐risk for structural anomaly following first trimester screening ultrasound, the comparison between referral first and second trimester ultrasound in continuing pregnancies yielded very good agreement in terms of presence or absence of fetal structural anomaly and a low rate of false positive and false negative diagnoses, the latter being most commonly represented either by minor structural anomalies with little or no impact in terms of pre‐ and perinatal management and counseling or by evolving anomalies that cannot be diagnosed in the first trimester. Consistently, most of the false positive diagnoses in the first trimester are consistent with the natural history of the identified structural defects, even though over‐diagnosis may be accounted as a potential limitation of first trimester referral ultrasound.

### 4.2 Comparison With and Discussion of Similar Research

Over the last decade great interest has arisen towards the potential of first trimester ultrasound in diagnosing fetal anomalies (Von Elm et al. 2014; Kagan et al. 2018; Chaoui and Nicolaides 2010). In 2011, Syngelaki et al. showed that at this gestational window fetal anomalies can be “always,” “never,” or “potentially” detectable depending upon the natural history of the disease, the expertise of the operator and ultrasound equipment (De Robertis and Italian Society of Ultrasound in Obstetrics and Gynecology (SIEOG) Working Group on the Obstetric Referral Scan 2023), however more recent data have suggested an increased frequency of early diagnosis of “potentially detectable” anomalies including open spina bifida, congenital heart defects and cleft lip and palate (Syngelaki et al. 2011). In such context, while several referral centers have reported variable detection rates for fetal anomalies (De Robertis and Italian Society of Ultrasound in Obstetrics and Gynecology (SIEOG) Working Group on the Obstetric Referral Scan 2023; Chaoui and Nicolaides 2010; Bardi et al. 2019; Liao et al. 2021), a meta‐analysis by Karim et al. demonstrated an almost two‐fold higher (61% vs. 32%) detection rate for all fetal structural anomalies in first‐trimester ultrasound in the context of a population at risk for fetal anomaly, compared with a low risk/unselected population (Karim et al. 2017). This may be dependent upon increased awareness of the ultrasound practitioners and greater technical expertise with focused study of the early fetal anatomy, machine setting and overcoming of acoustic‐window impairment (Kenkhuis et al. 2018; Bottelli et al. 2023).

The importance of the adoption of a formal anatomical screening protocol has been further confirmed by a recent meta‐analysis showing a higher overall detection rate for fetal anomalies, with the diagnoses of spina bifida, facial clefts and limb reduction being the most impacted by the introduction of a protocolized screening approach (Karim et al. 2024). Recently published data from a national program on fetal anomaly scan in the first trimester in a screening setting reported an overall sensitivity around 80% for “first trimester major congenital anomalies” (i.e., fetal anomalies can be “always” detectable in the first trimester) and 30% for all types of anomalies (Lust et al. 2024). Such figures may be explained by the (screening) clinical setting and by the criteria adopted to classify potentially evolving anomalies. In our study, the use of a standardized protocol for the assessment of the fetal anatomy has been associated with high sensitivity and specificity for fetal anomalies of first trimester referral ultrasound, and its agreement with referral ultrasound in the second trimester in non‐anomalous as well as in anomalous cases is higher if compared with the data reported in previous studies (Syngelaki et al. 2019; Liao et al. 2021).

In this research the reference to evaluate the performance of first trimester ultrasound was represented by second trimester referral ultrasound, which currently represents the gold standard for the expert assessment of the fetal anatomy (Rolnik, Wright, Poon, Syngelaki, et al. 2017; Carvalho et al. 2023; Lust et al. 2024). Such design of the study may have led to the exclusion of a significant number of anomalous cases with major structural defects or lethal anomalies undergoing termination of pregnancy, hence the inclusion of a selected cohort of cases with minor anomalies carrying less severe prognosis. Previous data support the increased detection rate of major structural anomalies compared with minor defects particularly if considering cardiac anomalies (Salomon et al. 2022; Kenkhuis et al. 2018; Sussman et al. 2021). In such context, most of the false negative diagnoses of first trimester referral ultrasound consist of minor cardiac defects including ventricular septal defect and persistent left superior vena cava. Of note, in such cases the fact that the diagnosis is most often made at later gestation is associated with little or no change in terms of prenatal counseling and management.

With respect to falsely positive diagnoses of first trimester referral ultrasound, most concern anomalies carrying the potential of spontaneous resolution with advancing gestation, which is the case of megacystis (van Nisselrooij et al. 2020), abdominal cyst (Fontanella et al. 2017), lymphangioma (Khalil et al. 2014; Graesslin et al. 2007), and Blake's Pouch cyst (Graesslin et al. 2007). However, in five cases a false positive diagnosis of major structural defects was made. This suggests a potential limitation of first trimester referral ultrasound as false positive diagnosis may be associated with anxiety and emotional consequences in the prospective parents (Chen et al. 2017).

### 4.3 Clinical and Research Perspectives

Early confirmation or exclusion of fetal anomalies in cases identified as at risk in a screening setting has been shown to improve clinical management, as delayed diagnosis may negatively impact maternal psychological well‐being and preclude less invasive options if termination of pregnancy is considered (Syngelaki et al. 2019; Chaoui and Nicolaides 2010; Gandolfi Colleoni et al. 2012). Early diagnosis allows sequential genetic investigations including exome sequencing (Lou et al. 2016) and multidisciplinary counseling for supporting informed decisions regarding pregnancy continuation versus termination. From this perspective, the findings from this study support the role of first trimester referral ultrasound for counseling and management of cases identified as at risk for anomaly following first trimester screening ultrasound: based on our data, a high degree of reassurance can be delivered to the patient in the event of a negative referral ultrasound scan, little changes in terms of prenatal counseling and management are expected in the event of “true” positive referral ultrasound scan, and also false negatives are to be accounted as minor defects carrying little impact in terms of counseling and management.

In this scenario, we believe that first trimester anatomy scan performed by experts according to a standardized protocol is feasible and has the potential to shift the timing of diagnosis of fetal anomaly in most cases. Therefore, it could be considered also in different healthcare settings on a cost‐effectiveness basis (Esteves et al. 2023; Mellis et al. 2022).

Conversely, looking at the potential drawbacks of first trimester referral scan, we reported three cases—that is, ~2% of the study population—of structural defects diagnosed in the first trimester, but not confirmed later in gestation. Regarding the false positive case of Blake's pouch cyst, little data from selected research groups exist on the evolving morphotype of the structures of the posterior fossa in the first and early second trimester (Pinto et al. 2014; Bak et al. 2020). Therefore, we believe that caution is warranted prior to reporting complex malformations of the posterior fossa in the first trimester (Volpe et al. 2021). Consistently, with respect to the case falsely diagnosed with tetralogy of Fallot, the reported detection rate of expert first trimester ultrasound for congenital heart defects has been shown to range between 56% in a low‐risk (Birnbaum et al. 2022) and 92% in a high‐risk setting at a single center (Debost‐Legrand et al. 2018), furthermore, the misdiagnosis of conotruncal anomalies has proven to be challenging also at later gestation (Karim et al. 2022; Haak et al. 2002; Tegnander et al. 2006). Finally, despite the improved detection rate of orofacial clefts over the last decades (Sander et al. 2024), the false negative diagnosis of cleft lip suggests that caution is warranted when diagnosing subtle anomalies as is a defect of the skin of the upper lip.

### 4.4 Strengths and Limitations

Our study is the first to report data on a systematic policy of referral first‐trimester ultrasound across referral Fetal Medicine Units following the publication of guidelines regulating the obstetric ultrasound practice on a national scale (Società Italiana di Ecografia Ostetrica e Ginecologia e Metodologie Biofisiche 2024; Italian Society For Ultrasound in Obstetrics and Gynecology (SIEOG) 2022). The criteria for patient selection and referral, the minimal standard in terms of ultrasound equipment and the univocal protocol for the systematic assessment of the fetal anatomy are among the strengths of the study, which was conducted across Fetal Medicine Units with expertise in the early diagnosis of fetal anomalies. The high agreement between first and second trimester referral ultrasound confirms previous data from single centers (Syngelaki et al. 2011), and the multicenter design of this study supports the generalizability of our findings.

With respect to the limitations, as per the design of this study the gold standard reference for anomalous vs. non‐anomalous fetal anatomy in the first trimester was represented by referral ultrasound scan performed in the second trimester, therefore information concerning third trimester ultrasound examination(s) (Kagan et al. 2008; Khalil et al. 2024), and pathology examination or magnetic resonance imaging (MRI) were not considered (Bilardo et al. 2023; Dolk et al. 2011; Prayer et al. 2023). As above, this might have led to the exclusion of a significant number of anomalous cases with major structural defects or lethal anomalies undergoing termination of pregnancy, and to the inclusion of a selected cohort of cases with minor anomalies carrying less severe prognosis. Previous data support the increased detection rate of major structural anomalies compared with minor defects particularly if considering cardiac anomalies (Salomon et al. 2022; Kenkhuis et al. 2018; Sussman et al. 2021). In such context, an undetermined number of unidentified anomalous cases (i.e., false negatives) submitted to prenatal ultrasound screening in the first trimester represented a population where the performance of first trimester referral ultrasound scan could not be investigated. However, it has to be acknowledged that a 22.5 rate of false negative results is reported also for midtrimester screening anomaly scan (Buijtendijk et al. 2024). Another limitation of the study is related to the nature of evolving structures as some false negative cases showed anomalies such as corpus callosum agenesis that cannot be diagnosed in the first trimester. Finally, we acknowledge also the absence of full postnatal examination of the cases alive at birth. However, the aim of this study was to compare what is new—that is, first trimester referral US—and what is meant to represent the gold standard for the assessment of the fetal anatomy—that is, second trimester referral ultrasound. In such context, it is worth underscoring that the existing guidelines (Salomon et al. 2022) endorse the role of prenatal ultrasound performed at midtrimester in antenatally suspecting (i.e., screening setting) or diagnosing (i.e., referral setting) fetal anomalies, which ultimately represents the reference for prenatal counseling and management.

## 5 Conclusion

In conclusion, we report excellent implementation of first‐trimester referral ultrasound in different Fetal Medicine units of the Italian territory in the 14 months following national Guidelines. First trimester referral ultrasound is feasible and has the potential to confirm or exclude a fetal anomaly in most cases. This allows pregnant women identified as high‐risk for fetal anomaly following first trimester screening ultrasound to promptly undergo necessary follow‐up investigations and take informed decisions regarding pregnancy management.

## Conflicts of Interest

The authors declare no conflicts of interest.

## Data Availability

The data that support the findings of this study are available on request from the corresponding author. The data are not publicly available due to privacy or ethical restrictions.
